# Study of the Effect of Retarder and Expander on the Strength and Cracking Performance of Rubber Concrete Based on Back Propagation Neural Network

**DOI:** 10.3390/ma16216976

**Published:** 2023-10-31

**Authors:** Chune Sui, Dan Qiao, Yalong Wu, Han Zhu, Haoyu Lan, Wenjun Yang, Qi Guo

**Affiliations:** 1 Department of Bridge Engineering, Transportation Institute, Inner Mongolia University, Hohhot 010070, China; sce2008_tl@163.com (C.S.); qd_0103@163.com (D.Q.); 2 China Railway Fourth Engineering Group Co., Ltd., Hefei 230041, China; 877300649@163.com; 3 Department of Civil Engineering, College of Architecture and Engineering, Tianjin University, Tianjin 300000, China; hanzhu2000@yahoo.com; 4 Tianjin Shiyi Urban Architectural Design Co., Ltd., Tianjin 300000, China; 13514792233@163.com; 5 Beijing ZZHX Engineering Research Institute Co., Ltd., Beijing 100000, China; duxingzhe_0_0@163.com

**Keywords:** rubber concrete, expander, retarder, compressive strength, flexural strength, crack resistance, BP neural network

## Abstract

The advantages of rubber concrete (RC) are good ductility, fatigue resistance, and impact resistance, but few studies have been conducted on the effects of different rubber admixtures on the strength of RC and the cracking performance of rubber mortar. In this study, the compressive and flexural tests of rubber concrete and the crack resistance test of rubber mortar were carried out by changing the rubber content and adding expansion agent and retarder in this test. The test results show that the strength of RC decreases with the increase in rubber admixture. At 15% of rubber admixture, the expansion agent and retarder increase the compressive strength and flexural strength of RC the most; the compressive strength increased to 116% (22.6 MPa) and 109% (21.2 MPa), and the flexural strength increased to 111% (4.02 MPa) and 116%. (4.21 MPa). At the same rubber admixture, the expander improves the cracking time of the rubber mortar by about 3 times, and the retarder improves the cracking time of the rubber mortar by about 1.6 times. The BP neural network (BPNN) was established to simulate and predict the compressive and flexural strengths of RC with different admixtures and the cracking time of rubber mortar. The simulation results show that the predicted 7-day compressive strength of RC fits best with the actual value, with a value of 0.994, and the predicted 28-day flexural strength was closest to the measured value, with an average relative error of 1.7%. It was shown that the calculation results of the artificial intelligence prediction model are more accurate. The simulation results and test results show that the expander and retarder significantly improve the strength of RC as well as the cracking performance of rubber mortar.

## 1. Introduction

With the rapid development of the economy and continuous optimisation of the building structure, the performance requirements of concrete are improving [1]. Rubber concrete (RC) refers to concrete with rubber particles as concrete components, and the study of rubber aggregate concrete began in the 1990s and has achieved certain results. Xue Gang et al. [2] studied the effect of rubber powder admixture on the compressive properties of RC, in which 5%, 10%, 15%, and 20% of rubber were admixed into the concrete to make 100 mm × 100 mm × 300 mm prismatic specimens, and it was found that the ductility of RC was greatly enhanced and the energy dissipation was significantly increased compared with that of ordinary concrete. Su-Rong Luo et al. [3] measured the load–displacement curves and crack opening displacements of three-point bending and tensile specimens and applied the double-K model of fracture mechanics to calculate the fracture energy and fracture toughness, and the results showed that the fracture toughness of RC was significantly improved, and in addition to the rubber particle size and admixture amount, the treatment of the rubber surface would also have an effect on the concrete toughness. Fu Qian et al. [4] conducted uniaxial compression tests of RC with water–cement ratio, rubber admixture, rubber particle size, and fly ash admixture as the influencing factors and found that the whole damage process of RC was more moderate than that of ordinary concrete. Meanwhile, the strength of the concrete was reduced due to the addition of rubber particles, but the ductility, toughness, and energy dissipation capacity were improved. Mezidi Ama et al. [5] studied the compressive strength of RC with rubber replacement rates of 1%, 2%, 3%, 4%, and 5% and obtained that the concrete with 3% rubber admixture performed better in terms of compressive strength. Ayman Abdelmonem et al. [6] conducted an in-depth study on RC slump, compressive strength, tensile strength, and flexural strength using rubber with 0%, 10%, 20%, and 30% partial substitution of fine aggregate volume and obtained that the addition of rubber changed the concrete compatibility, but as the rubber admixture reached 30%, the concrete compressive and tensile strength values of the concrete showed a significant decrease of 50%. According to a large number of scholars at home and abroad on the physical and mechanical properties of RC [7,8,9,10,11], found that compared with ordinary concrete, RC has good elasticity, toughness, freeze–thaw resistance, superior deformation properties, and crack resistance; these properties could make up for the poor elastic deformation of ordinary concrete, lack of fatigue resistance, poor impact resistance, and other defects. In addition, RC is used in civil construction because of its superior ductility, toughness, impact resistance, and other properties, and it also has a wide application space in airport runways, impact-resistant guardrails, and some military buildings. At the same time, RC has become one of the most important ways to reuse waste rubber tires, which not only can reduce the environmental pollution problem caused by waste rubber tires but also improve the performance of concrete [12].

In recent years, expansion agents and retarders have been widely used in various fields of practical engineering. Expansion agents play an important role in both compensating shrinkage and generating self-stress; compensating shrinkage could reduce or even avoid the cracking of concrete due to volume deformation, and adding expansion agents to concrete shows good durability performance [13]. The retarders could significantly delay the exothermic rate of cement hydration reaction, thus reducing the temperature cracks in concrete due to concentrated exothermic hydration and possibly effectively extending the setting time of concrete and improving the efficiency of concrete use [14,15,16]. Many scholars have conducted a lot of research based on this. Chen Bo et al. [17] studied the compound effect of an internal curing and expansion agent using the temperature test method and found that the compound effect of an internal curing agent and expansion agent is significant for improving the crack resistance of concrete. Shen Dejian et al. [18] studied the effect of the MgO compound expansion agent (MCEA) on the early cracking damage behaviour of concrete. Litina Chrysoula et al. [19] evaluated the effectiveness of the proposed test method applicable to self-healing concrete doped with expansive minerals. Jinjun Guo et al. [20] investigated the effect of the combined expansive agent (UEA) and MgO expansive agent on the fracture performance of concrete through a three-point bending test. Hao Lei et al. [21] found that the ability of the expander to enhance the plastic cracking resistance of concrete was related to the water–cement ratio of concrete, and the effect of the expander to enhance the plastic cracking resistance of concrete decreased with the decrease in the water–cement ratio and even had a negative effect. A.W. Otunyo et al. [22] investigated the application of urea fertiliser (UF) as a slow setting admixture in plain concrete. Sutarto Tommy Ekamitra et al. [23] experimentally evaluated the performance of sucrose and lignosulfonic acid as concrete retarders and obtained the initial and final setting times of concrete with different admixture doses. In addition, the effect of retarders on the compressive strength of concrete was also explored. Wang Lijiu et al. [24] used the absolute warming method to determine the exothermic process of the new super retarder on the heat of the hydration of bulk concrete and discussed the hydration kinetics and the effect on early thermal cracking. Xu Changwei et al. [25] used three retarders, borax, citric acid, and sodium gluconate, to study the effect of different retarders on C50 low-chloride concrete and found that the addition of retarders could effectively reduce the time-loss of concrete, while reducing the early volume deformation of concrete, and had no adverse effect on the later strength.

In order to save time, cost, and improve material performance, researchers have predicted the strength of materials based on different machine learning models; the most commonly used method is the back propagation neural network (BPNN). The most important features of artificial neural networks are collective computing, adaptive, excellent fitting ability to highly nonlinear insinuations, and fault tolerance to sample learning. The network can make full use of state information and train the information from different states one by one to obtain balanced convergence weights, which represent the nonlinear mapping relationship of the network. Therefore, it could well find the intrinsic relationship between influencing factors. Han et al. [26] used the BPNN to analyse the effect of different admixtures of waste fly ash on the compressive strength of high-performance concrete, and the results showed that the BPNN has high accuracy in predicting the compressive strength of waste fly ash concrete. Du Huanhuan et al. [27] established a three-dimensional finite element numerical analysis model for the flexural performance of prestressed steel–concrete continuous composite beams and simulated the whole process of the test based on the BPNN. Cao et al. [28] proposed a fully convolutional neural network to automatically identify and detect cracks by constructing a framework for learning concrete cracks and performing semantic segmentation to achieve crack identification. The method was found to be able to accurately identify crack paths and crack density variations through validation. Liu Zhenhua et al. [29] proposed a novel method for predicting roadbed settlement by combining fuzzy neural networks and wavelet packet decomposition to establish a settlement prediction model by using the powerful memory of fuzzy neural networks for the temporal and accidental nature of displacement settlement sequences. Sadrmomtazi et al. [30] compared the accuracy of predicting concrete strength using regression analysis, the neural network model, and fuzzy algorithm, and the results showed that the neural network model has a higher accuracy and generalisation ability.

In summary, there have been a large number of studies on RC, concrete mixed with expanders and retarders, but there are very few studies on the compound effect of adding expanders and retarders to RC, and the prediction of RC using the BPNN is less common in studies on RC. Therefore, this paper investigates the strength of plain concrete, rubber aggregate concrete, rubber aggregate concrete with expander and retarder, and the cracking resistance of plain mortar, rubber aggregate mortar, and rubber aggregate mortar with expander and retarder. Meanwhile, the prediction models of compressive strength, flexural strength, and cracking resistance of rubber aggregate mortar with multiple parameters such as cement, sand, and stone acting simultaneously were established by combining the BPNN approach in order to provide theoretical support for the subsequent research on RC.

## 2. Materials and Experiments

### 2.1. Material

Cement adopts Camel brand P-O42.5 ordinary silicate cement, and its main technical properties are shown in Table 1; fine aggregate adopts ordinary natural river sand with a fineness modulus of 2.9 and apparent density of 2670 kg/m^3^; rubber granule adopts rubber with an average particle size of 3 mm produced by Tianjin Kewei Rubber Factory (Tianjin, China) and an apparent density of 1050 kg/m^3^, the specific technical indicators are shown in Table 2; coarse aggregate adopts gravel with a particle size of 5–20 mm and apparent density of 2680 kg/m^3^; water is ordinary tap water; the expansion agent adopts the UEA-6 concrete expansion agent produced by Beijing Muhu Company (Beijing, China); and the retarder adopts the concrete retarder produced by Hebei Qingjun Chemical Factory (Cangzhou, China).

### 2.2. Mix Design

The ordinary concrete, the 5%, 10%, and 15% rubber single admixture of concrete (in which rubber replaced sand and gravel at an equal volume), the 6% expander admixture, with 5%, 10%, and 15% rubber admixture double admixture of concrete (in which the expander replaced cement by an equal mass), and the 0.1% retarder admixture, with 5%, 10%, and 15% rubber admixture double admixture of concrete (in which the retarder replaces cement by an equal mass) were studied in this paper when measuring the influence of adding an expansion agent and retarder on the mechanical properties. The mix ratio is shown in Table 3. The PM was ordinary concrete. The RM5 was concrete with 5% rubber content. The RM5P6 was concrete with 6% expansion agent content and 5% rubber content. The RM5H was concrete with 0.1% retarder content and 5% rubber content, and so on.

Mortar was used as the object of study in determining the effect of the admixture of the expander and retarder on the cracking resistance of RC due to the similarity between the cracking properties of concrete and mortar. The ordinary mortar, the 5%, 10%, and 15% rubber-doped mortar (in which rubber was replaced by an equal volume of sand), the 5% expander dosing, with 5%, 10%, and 15% rubber-doped mortar (in which the expander was replaced by an equal mass of cement), and the 0.1% retarder dosing, with 5%, 10%, and 15% rubber-doped mortar (in which the retarder was replaced by an equal mass of cement) were studied in the paper. The mix ratio is shown in Table 4. The PM was ordinary mortar. The RM5 was mortar with 5% rubber dosing. The RM5P5 was mortar with 5% expansion agent dosing and 5% rubber dosing double blending. The RM5H was mortar with 0.1% retarder dosing and 5% rubber dosing double blending, and so on.

## 3. Test Method

### 3.1. Determination of Compressive Strength and Flexural Strength of Rubber-Expanded Concrete

The test was conducted according to GB/T50081-2002 ‘Test Methods for Mechanical Properties of Ordinary Concrete’ [31]. There were 10 proportions and 3 test pieces for each proportion in the test. The compressive strength and flexural strength were tested, respectively, after the specimens were formed in accordance with the standard curing method to 7 days and 28 days.

The steps of the compression test are as follows:

(1) The specimen with a size of 100 mm × 100 mm × 100 mm was placed on the lower plate of the testing machine. The centre of the specimen was aligned with the centre of the lower plate of the testing machine, and the testing machine was started.
(2) The ball seat was adjusted to make the contact balanced when the upper pressure plate was close to the specimen. The load was continuously and evenly loaded during the test.
(3) We stopped adjusting the accelerator of the test machine when the specimen was close to failure and began to deform sharply until the specimen was destroyed. We also recorded the failure load.

The test steps of flexural strength test are as follows:

(1) The 100 mm × 100 mm × 400 mm specimen was installed on the press, and the bearing surface of the specimen was the side of the specimen when formed.
(2) The support and the pressure surface were in smooth and uniform contact with the cylinder, and the uniform and continuous load was applied.
(3) The accelerator of the testing machine was stopped when the specimen was close to failure until the specimen was destroyed. Then, we recorded the indication of the failure load testing machine and the fracture position of the lower edge of the specimen.

### 3.2. Mortar Crack Resistance Test

**Figure 1:**
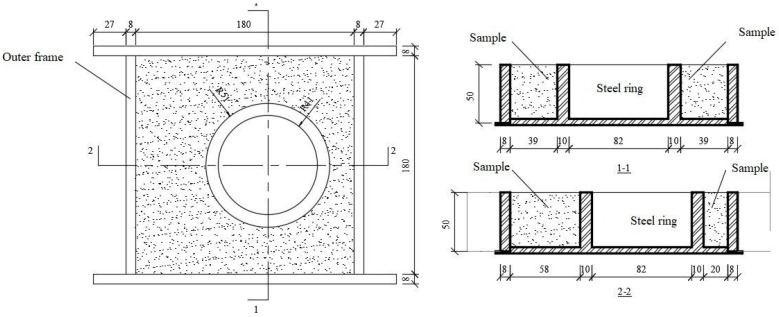
Mould shape and size (unit: mm).

The external square inner circle eccentric constraint method and internal circle for measurement were used in the test to measure the mortar cracking time, which could predict the approximate location of cracks and facilitate the observation of cracks. There were 10 proportions and 6 specimens of each proportion in the test. The external square inner circle eccentric constraint method shortened the cracking time of the specimen, improved the test efficiency, and shortened the cycle time. The shape and dimensions of the mould are shown in Figure 1.

**Figure 2:**
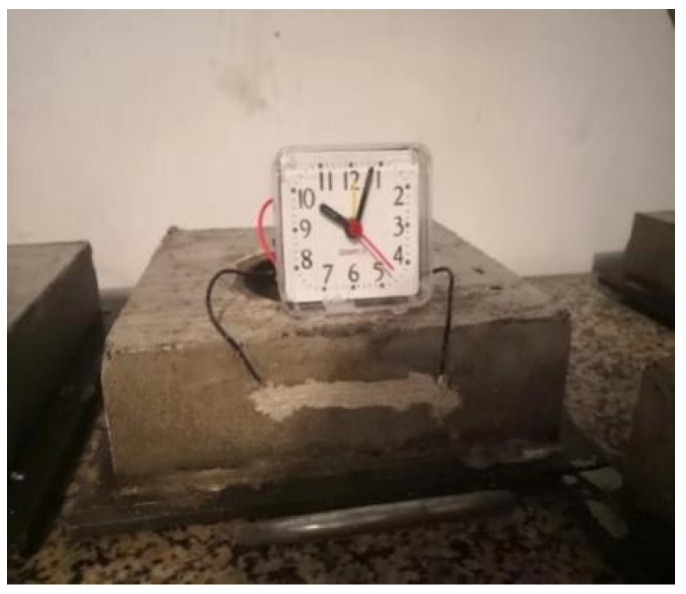
Automatic detection device.

The test steps of the crack resistance test are as follows:

(1) The test mould was cleaned and the inner wall was coated with oil before the test.
(2) The mortar was poured into the test mould and immediately put into the curing room after forming. The surface was wiped twice to facilitate the observation of cracks after 6 h.
(3) Crack observation equipment was installed after removing the mould, as shown in Figure 2. A layer of conductive adhesive was coated near the cracking position and connected with the alarm clock by wire to form a closed path.
(4) The alarm clock time was adjusted to the current time and recorded every 12 h to observe whether the alarm clock stopped. Each group of specimens was observed continuously for 30 days.

## 4. Test Results and Discussion

### 4.1. Determination of Compressive and Flexural Strength of RC

**Figure 3:**
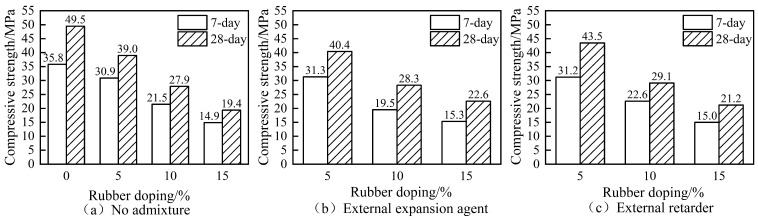
Seven-day and twenty-eight-day compressive strength of rubber concrete (RC).

The compressive strength graphs at 7-day and 28-day ages for concrete without admixture, with the expander and retarder at different rubber admixtures, are shown in Figure 3.

The compressive strength of concrete decreases with the increase in rubber admixture whether at 7 days or 28 days, as shown by Figure 3a. The compressive strength of RC decreased to about 40% of the strength of ordinary concrete when the rubber admixture was 15%. In addition, the admixture of rubber provided the concrete with a certain early strength effect, and the compressive strength at 7 days already reached about 78% of that at 28 days. The compressive strength of rubber–intumescent concrete decreases with an increasing rubber admixture in the case of an additional expansion agent, as shown by Figure 3b. The compressive strength of rubberised concrete increased at both 7 days and 28 days after the admixture of the expansion agent compared Figure 3a,b. The 28-day compressive strength increased by 116% when the rubber admixture was 15%. The compressive strength of rubber-retarded concrete decreased with the increase in the rubber admixture in the case of additional retarder, as shown by Figure 3c. The compressive strength of RC at 7 days and 28 days also increased after the addition of retarder, and the 28-day compressive strength increased by 109% when the rubber admixture was 15% compared Figure 3a,c. Overall, the amount of rubber admixture had a significant effect on the compressive strength of concrete, which decreased continuously as the amount of rubber admixture increased [6], and the expansion agent and retarder had little effect on the compressive properties of rubber concrete at small admixtures, which was the same as the conclusions reached in the literature [32,33].

The main reason for the reduction in the compressive strength of RC was the different material properties between rubber particles and cement paste; the rubber used was a polymer organic compound, while cement paste was inorganic. The bond strength between rubber particles and cement paste was low then the rubber particles and cement paste could not be bonded together very effectively. The impact on the strength after the combination of rubber and the expansion agent was the common result of multiple factors. Besides the fact that the rubber and concrete could not be well combined, the other reasons were that, firstly, the admixture of rubber introduced a certain amount of pores inside the concrete, and the more the admixture introduced the larger the number of closed pores there were; the addition of the expansion agent could have made the concrete more dense, thus enhancing the strength of the concrete. Secondly, the rubber itself could reduce the stress concentration caused by the excessive expansion agent to a certain extent and reduce the number of expansion agent micro-cracks when the expansion agent was too large, thus affecting the strength of the rubber-expanded concrete. The impact of the rubber and retarder combination on strength was also caused by multiple factors: in addition to the fact that the rubber and concrete could not be effectively combined, part of the reason was that the main role of the retarder was to extend the setting time of the concrete. The temperature cracks was caused by the hydration heat. The retarder added in the concrete can enhance the strength of the concrete and achieve the effect of low-volume super-retardation which can reduced the temperature cracks caused by the hydration heat of the concrete.

**Figure 4:**
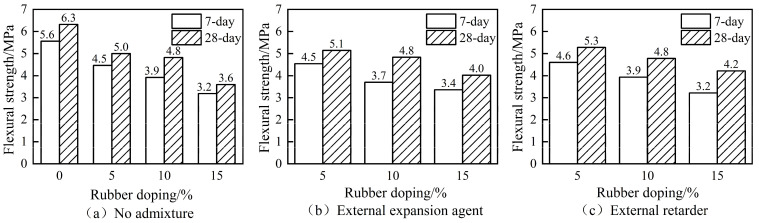
Seven-day and twenty-eight-day flexural strength of RC.

The flexural strength of concrete without the admixture, with the expander, and with the retarder at 7 days and 28 days for different rubber admixtures is shown in Figure 4.

The flexural strength of concrete decreased with the increase in the rubber admixture, as shown by Figure 4a, both at 7 days and 28 days. The flexural strength of RC decreased to about 57% of the strength of ordinary concrete when the rubber admixture was 15%. The early strengthening effect of rubber in RC was also reflected in the flexural strength data, and the 7-day flexural strength reached 89% of the 28-day flexural strength when the rubber admixture was 5%. The flexural strength of rubber-expanded concrete also decreased with the increase in the rubber admixture in the case of an additional expansion agent, as shown by Figure 4b. The compressive strength of rubberised concrete increased at both 7 days and 28 days after the addition of the expansion agent, compared to Figure 4a,b, and the 28-day flexural strength increased by 111% when the rubber admixture was 15%. The flexural strength of rubber-retarded concrete also decreased with the increase in the rubber admixture in the case of an additional retarder, as shown by Figure 3c. The flexural strength of RC increased at both 7 days and 28 days after the addition of the retarder, compared to Figure 4a,c, and the 28-day flexural strength increased by 116% when the rubber admixture was 15%. Overall, the amount of rubber admixture had a significant effect on the flexural strength of the concrete, and the expansion agent and retarder had little effect on the flexural performance of RC at small admixtures. The main reason for the decrease in the flexural strength of RC was the same as the analysis of compressive strength.

### 4.2. Determination of Mortar Cracking Time

The cracking times of different mortars are shown in Table 5.

The addition of rubber-retarded the cracking of mortar, as shown by Table 5, and the cracking time of mortar increased with the increase in the rubber admixture. When the rubber admixture reached 15% of the total volume, the cracking time reached 137% of that of ordinary concrete. The admixture of expansion agent and retarder can delay the cracking time of rubber mortar and improve the cracking resistance of rubber mortar [33], in which the expansion agent shows a superior performance in cracking resistance and the double admixture of rubber and an expansion agent doubly improves the cracking resistance of mortar after the cracking time of rubber expansion mortar is greatly delayed. The average cracking time increased by about three times with the same amount of rubber, and both the initial cracking time and average cracking time were improved with the increase in rubber dosing. The retarder also improved the cracking resistance of RC, the same rubber admixture, and rubber-retarded concrete, compared to the RC average cracking time which increased by about 1.6 times, and both the initial cracking time and average cracking time require an increase in the rubber admixture in order to improve.

The two reasons for the occurrence of non-structural cracking were mortar shrinkage and mortar brittleness. Introducing anticracking components that abated the two factors in order to improve the crack resistance of mortar was necessary. The reason why mixing rubber was effective in delaying cracking is inseparable from the properties of rubber granules themselves. Rubber granules are a flexible material that can improve the brittleness of concrete and produce larger deformation after internal stress, which could relieve the stress concentration phenomenon and enhance the deformation resistance of concrete. When shrinkage deformation occurred, part of the shrinkage stress was directly absorbed by the internal rubber granules, which could also block the water seepage channels and slow down the rate of drying and evaporation of water, thus delaying the appearance of cracks [34]. The swelling agent is a more widely used anticracking component, and the appropriate amount of swelling agent could compensate for the shrinkage of the mortar due to various reasons, reduce the shrinkage stress, and delay the cracking of the mortar. After the double admixture of the expander and rubber, the cracking of mortar was inhibited from both shrinkage and brittleness, and the cracking resistance of mortar was improved. The retarder could delay and reduce the peak of the heat of hydration, thus achieving the effect of delaying the appearance of early temperature cracks, and could reduce the number and width of internal voids and surface cracks of mortar. In addition, the addition of retarder could effectively improve the hydration efficiency of cement and make the internal product distribution of mortar more uniform while reducing the number of structural defects. Similar to the action principle of rubber expansion mortar, the double admixture of retarder and rubber suppressed both shrinkage and brittleness factors under the joint action of both and improved the crack resistance of mortar.

## 5. BPNN Prediction Model

### 5.1. Modelling

The BPNN is a multilayer perceptron based on error back propagation and usually consist of an input layer, an implicit layer, and an output layer substructure [35]. In this study, cement, sand, gravel, water, rubber, expander, retarder, and RC compressive strength, flexural strength, and mortar cracking time were selected as input vectors and test values to establish an RC strength prediction model based on the BPNN.

MATLAB is a language software based on network theory, and its main function is to deal with problems between data computation and computer simulation, where the neural network toolbox is a typical network tool function. It is extremely easy and fast to build neural networks using MATLAB [36]. The BPNN prediction model was established in this study with the help of the neural network toolbox provided by MATLABR2018b, and the establishment process is as follows:

(1) Identify the input, implicit, and output layers.

**Figure 5:**
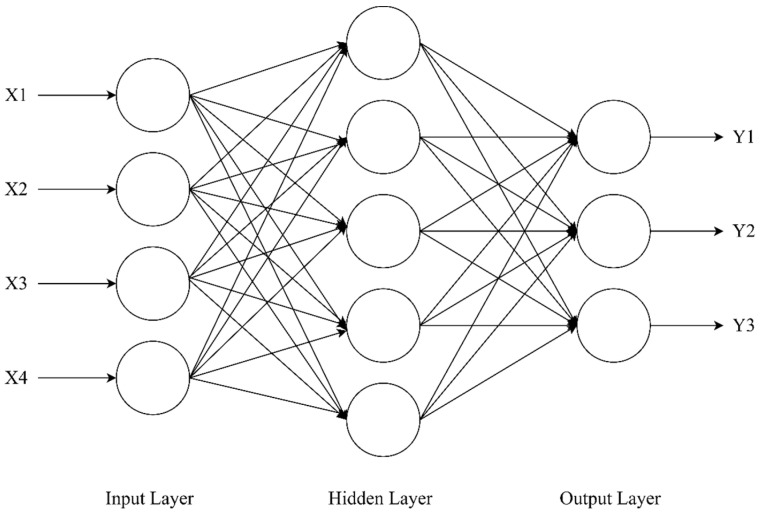
BP neural network (BPNN) structure.

The typical structural composition of the BPNN consists of three layers: the input layer, the hidden layer, and the output layer. The structure is shown in Figure 5.

**Figure 6:**
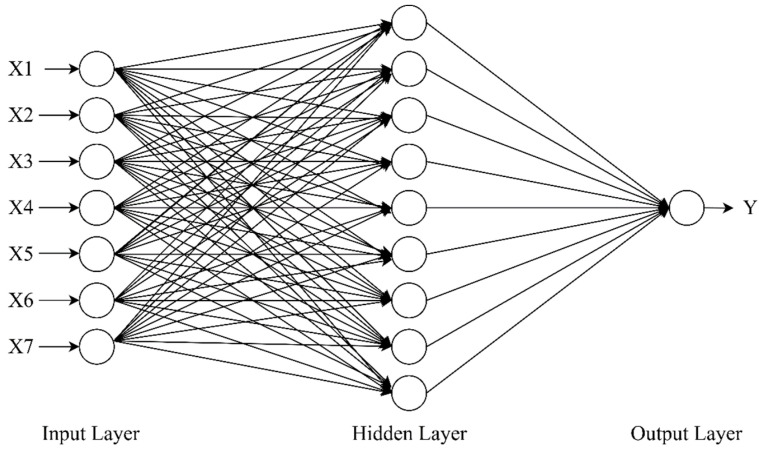
Schematic diagram of the structure of the BPNN.

The input vectors were fed through the input layer and then processed by the implicit layer and passed out through the output layer [36]. The structure of the BPNN prediction model established in this study was 7-9-1, where 7 indicated that the number of nodes in the input layer was 7, 9 indicated that the number of nodes in the hidden layer was 9, and 1 indicated that the number of nodes in the output layer was 1. The structure diagram of the BPNN model is as follows (Figure 6):

(2) Selection of samples and normalisation of sample data.

The samples in this study were selected from the data obtained from the previous tests on the compressive strength of RC, flexural strength, and rubber mortar cracking time. In order to obtain accurate simulation results, 100% of the sample data were selected as the training sample as well as the test sample. The sample data were normalised to map the sample data to the interval of [0,1] or [−1,1] or other intervals to accelerate the convergence of the neural network and reduce the training time. The data normalisation algorithm is as follows:

(1) $$y=\frac{x-min}{max-min}$$

(2) $$y=2\times \frac{x-min}{max-min}-1$$

(3) Establishing the BPNN. The two focal functions in the establishment process are:
  - mapminmax: process the matrix by mapping the minimum and maximum values to [−1 1]; the expression and related parameters are explained as follows:

(3) [Y,PS]=mapminmax(X,YMIN,YMAX)

(4) [Y,PS]=mapminmax(X,FP)

(5) Y=mapminmax(‘apply’,X,PS)

(6) X=mapminmax(‘revers’,Y,PS)

(7) dx_dy=mapminmax(‘dx_dy’,X,Y,PS)

The mapminmax processes matrices by normalizing the minimum and maximum values of each row to [YMIN, YMAX]. Then, [Y, PS] = mapminmax (X, YMIN, YMAX) takes X and optional parameters. Then, X means N-by-Q matrix, YMIN means Minimum value for each row if Y (default is −1), and YMAX means Maximum value for each row of Y (default is +1).

b. newff, creating a feed forward back propagation network with the following function expression:

(8) net = newff (P, T, [T1, S2…S (N−1)], {TF1, TF2…TFNI}, BTF, BLF, OPF, DDF)

TFi means the Transfer function of the ith layer. Default is ‘tansig’ for hidden layers, and ‘purelin’ for output layer. BTF means Backprop network training function, default = ‘trainlm’. BLF means Backprop weight/bias learning function, default = ‘learngdm’. PF means Performance function, default = ‘mse’. IPF means Row cell array of input processing functions. Default is {‘fixunknowns’, ‘remconstantrows’, ‘mapminmax’}. OPF—Row cell array of output processing functions. Default is {‘remconstantrows’, ‘mapminmax’}. DDF means Data division function, default = ‘dividerand’.

(4) BPNN parameters setting. In this study, the number of iterations of the designed neural network was 0–1000; the training target error was set to 10-4; and the learning rate was set to 0.01.

In this study, the evaluation of the prediction results of the BPNN model relies on the relative error and the coefficient of determination R^2^. The relative error (error) values and the coefficient of determination R^2^ calculated from the BPNN model are shown in Table 6, Table 7 and Table 8. The formula for calculating the relative error is as follows:

(9) $$\delta =\frac{∆}{L}\times 100\%$$

R^2^ was calculated as follows:

(10) $$R^{2}=1-\frac{{\sum (y-y^{⋀})}^{2}}{\sum {\left(y-\bar{y}\right)}^{2}}$$

Among them, $∆=T_{-test}-T\_sim$, L = T_test.

**Table 6:** Predicted values of compressive strength of concrete and evaluation coefficients calculated by BPNN model.

7-Day Compressive Strength, R^2^ = 0.9944			28-Day Compressive Strength, R^2^ = 0.9877		
T_sim	T_test	Error%	T_sim	T_test	Error%
35.79	35.8	0.0004	48.98	49.5	0.0106
30.00	30.9	0.0290	39.00	39.0	0.0001
22.27	21.5	0.0356	27.90	27.9	0.0002
14.85	14.9	0.0031	19.41	19.4	0.0005
31.54	31.3	0.0076	40.39	40.4	0.0001
19.86	19.5	0.0186	30.42	28.3	0.0750
15.28	15.3	0.0012	22.60	22.6	0.0000
31.26	31.2	0.0020	43.50	43.5	0.0000
22.37	22.6	0.0101	32.83	29.1	0.1283
16.52	15.0	0.1014	21.26	21.2	0.0029
Average Error/%		0.0209	Average Error/%		0.0218

**Table 7:** Predicted values of flexural strength of concrete and evaluation coefficients calculated by BPNN model.

Day Break off Strength, R^2^ = 0.9863			28-Day Break off Strength, R^2^ = 0.9792		
T_sim	T_test	Error%	T_sim	T_test	Error%
5.56	5.56	0.0008	6.32	6.32	0.0002
4.63	4.46	0.0382	4.46	5.00	0.0001
3.94	3.92	0.0043	3.94	4.82	0.0049
3.28	3.19	0.0280	3.46	3.60	0.0857
4.57	4.54	0.0057	5.14	5.14	0.0001
3.73	3.70	0.0085	4.64	4.83	0.0397
3.35	3.36	0.0038	4.02	4.02	0.0006
4.73	4.60	0.0284	5.03	5.28	0.0480
3.95	3.93	0.0046	4.78	4.78	0.0001
3.02	3.22	0.0622	4.21	4.21	0.0002
Average Error/%		0.0184	Average Error/%		0.0180

**Table 8:** Predicted values of mortar cracking time and evaluation coefficients calculated after BPNN model.

Cracking Time of Initial Cracking Specimen/h, R^2^ = 0.9912			Average Cracking Time/h R^2^ = 0.9743		
T_sim	T_test	Error%	T_sim	T_test	Error%
85.69	86	0.0036	83.93	90	0.0675
78.60	97	0.1897	120.56	101	0.1937
95.66	110	0.1304	135.37	115	0.1771
114.10	118	0.0330	138.27	128	0.0802
269.66	263	0.0253	304.30	300	0.0143
314.24	338	0.0703	366.17	363	0.0087
378.73	385	0.0163	406.94	412	0.0123
149.73	145	0.0326	200.95	164	0.2253
164.86	167	0.0128	187.56	185	0.0138
179.99	183	0.0165	164.58	196	0.1603
Average Error/%		0.0530	Average Error/%		0.0953

### 5.2. Analysis of Prediction Results

**Figure 7:**
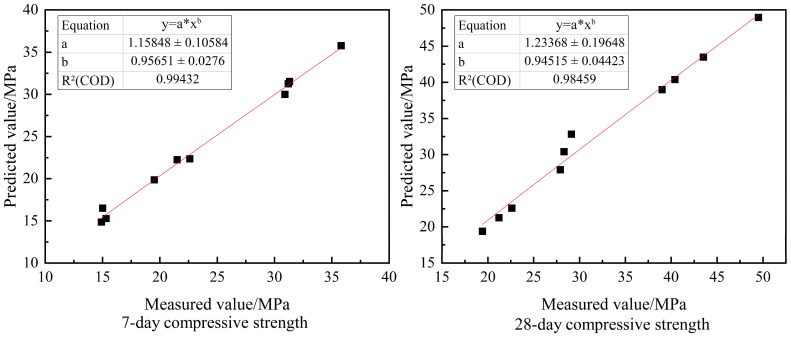
Scatter plot of measured and predicted compressive strength values.

The predicted values of 7-day and 28-day compressive strength of RC were obtained according to the results of the BPNN model operation, and the predicted values were compared with the measured values measured by the test; the errors between the predicted values of 7-day and 28-day compressive strength and the real values were concentrated within 2%, and the prediction accuracy was high. The two were fitted, as shown in Figure 7. The R^2^ values of both reached 0.98, which showed a good fitting effect.

**Figure 8:**
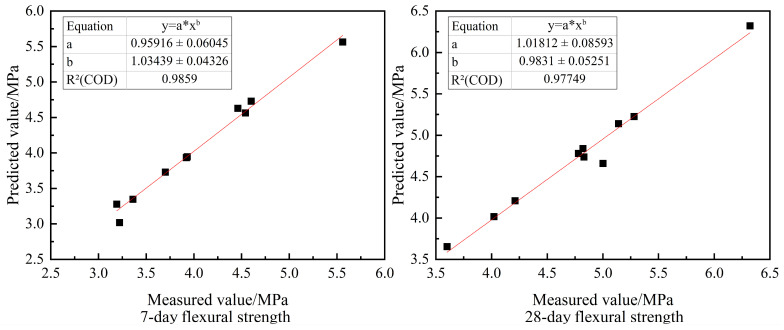
Scatter plot of measured and predicted flexural strength values.

The predicted values of the 7-day and 28-day flexural strength of RC were obtained according to the results of the BPNN model operation, and the predicted values were compared with the measured values measured in the test. The 7-day and 28-day flexural predicted values were concentrated within 2% error from the real values, and the prediction accuracy was high. The two were fitted as shown in Figure 8. The R^2^ values of both reached 0.97, showing a good fitting effect.

**Figure 9:**
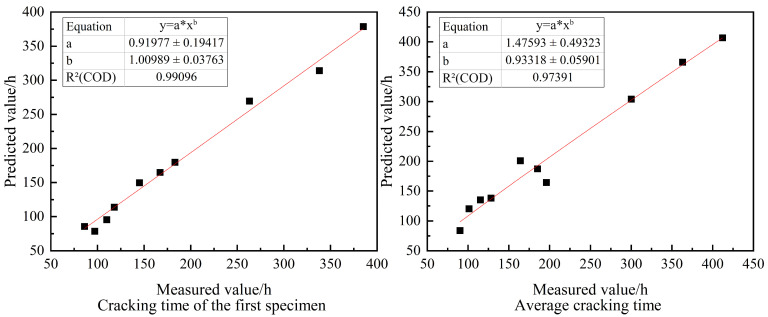
Scatter plot of measured and predicted cracking time values.

The predicted values of cracking time and average cracking time of the first specimen of rubber mortar were obtained according to the results of the BPNN model run, and the predicted values were compared with the actual values measured by the test; the errors of the predicted values of cracking time and average cracking time of the first specimen and the real values were concentrated within 10%, and the prediction accuracy was high. The two were fitted as shown in Figure 9. Both R^2^ values reached 0.97, which shows a good fitting effect.

The prediction model established by the BPNN is much better than the previous prediction by fitting the traditional empirical formula and again verifies the high accuracy of the artificial neural network prediction model, which has established a relationship between the amount of cement, sand, stone, water, rubber, expander, retarder admixture, and the compressive strength, flexural strength, and cracking time coefficient of rubber mortar. Given the amount of cement, sand, gravel, water, rubber, expander, and retarder, the compressive strength, flexural strength, and cracking time of the formulated RC could be predicted by substituting the artificial neural network. The use of neural network to predict the compressive strength, flexural strength, and cracking time of RC was not only efficient, but it also avoids the waste of human, material, and financial resources caused by conventional formulation tests.

## 6. Conclusions and Recommendations

The compressive and flexural strength of RC decreased with the increase in the rubber admixture. The compressive and flexural strength had different degrees of growth with the addition of an expander and retarder, rubber-expanded concrete, and rubber-retarded concrete compared to RC. The compression–folding ratio of concrete was reduced to make up for the reduction in concrete strength due to the addition of rubber at the same time.

The single admixture of rubber in mortar improved the crack resistance of mortar, but the improvement was not great. After the addition of the expander and retarder, the anticracking property of rubber mortar was doubly improved under the joint action of admixture and rubber.

The training model based on the BPNN accurately predicted the compressive and flexural strength of RC as well as the cracking time of rubber mortar, where the values reached above 0.97 with a high prediction accuracy. The BPNN showed good applicability to the RC strength prediction problem and could obtain the material proportioning that meets the engineering requirements quickly and accurately verified through the test.

## Figures and Tables

**Table 1:** Main technical properties of cement index.

Specific Surface Area (m^2^/kg)	Setting Time (h)		Compressive Strength (MPa)			Flexural Strength (MPa)		
	Initial Setting Time	Final Setting Time	3 d	7 d	28 d	3 d	7 d	28 d
346	2:07	5:20	36.2	35.7	52.2	3.9	5.4	7.9

**Table 2:** Technical specifications of rubber granules.

Material	Average Particle Size (mm)	Sieve Number (mesh)	Sieving Rate (%≥)	Moisture Content (%≤)	Ash Content (%≤)	Acetone Extraction (%≤)	Fiber Content (%≤)	Metal Content (%≤)
Rubber Granules	3.0	15	95	1.0	1.0	15	0.5	0.08

**Table 3:** Different doping rubber strength determination ratio.

Experimental Group Number	Cement (kg)	Sand (kg)	Stone (kg)	Water (kg)	Rubber (kg)	Expansion Agent (kg)	Retarder (kg)
PM	439	629	1118	215	0	0	0
RM5	439	581	1032	215	36	0	0
RM10	439	532	946	215	72	0	0
RM15	439	484	860	215	108	0	0
RM5P6	413	581	1032	215	36	26.3	0
RM10P6	413	532	946	215	72	26.3	0
RM15P6	413	484	860	215	108	26.3	0
RM5H	439	581	1032	215	36	0	0.43
RM10H	439	532	946	215	72	0	0.43
RM15H	439	484	860	215	108	0	0.43

**Table 4:** Crack resistance measurement ratio of rubber mortar with different dosing.

Experimental Group Number	Cement (kg)	Sand (kg)	Water (kg)	Rubber (kg)	Expansion Agent (kg)	Retarder (kg)
PM	720	1080	360	0	0	0
RM5	720	948	360	22	0	0
RM10	720	815	360	44	0	0
RM15	720	682	360	66	0	0
RM5P5	684	948	360	22	36	0
RM10P5	684	815	360	44	36	0
RM15P5	684	682	360	66	36	0
RM5H	720	948	360	22	0	0.7
RM10H	720	815	360	44	0	0.7
RM15H	720	682	360	66	0	0.7

**Table 5:** Cracking time of rubber mortar with different dosing.

Specimen Group Number	Cracking Time of Initial Cracking Specimen/h	Average Cracking Time/h
PM	86	90
RM5	97	101
RM10	110	115
RM15	118	128
RM5P5	263	300
RM10P5	338	363
RM15P5	385	412
RM5H	145	164
RM10H	167	185
RM15H	183	196

## Data Availability

All data that support the findings of this study are available from the corresponding author upon reasonable request.

## Nomenclature

mapminmax	Processes matrices by normalizing the minimum and maximum values of each row to [YMIN, YMAX].
[Y, PS]	mapminmax (X, YMIN, YMAX): takes X and optional parameters.
X	N-by-Q matrix.
YMIN	Minimum value for each row if Y (default is −1).
YMAX	Maximum value for each row of Y (default is +1).
TFi	Transfer function of ith layer. Default is ‘tansig’ for hidden layers, and ‘purelin’ for output layer.
BTF	Backprop network training function, default = ‘trainlm’.
BLF	Backprop weight/bias learning function, default = ‘learngdm’.
PF	Performance function, default = ‘mse’.
IPF	Row cell array of input processing functions. Default is {‘fixunknowns’, ‘remconstantrows’, ‘mapminmax’}.
OPF	Row cell array of output processing functions. Default is {‘remconstantrows’, ‘mapminmax’}.
DDF	Data division function, default = ‘dividerand’.
